# Development and validation of the Terminal Delirium-Related Distress Scale – Shortform

**DOI:** 10.1017/S1478951525000227

**Published:** 2025-03-14

**Authors:** Megumi Uchida, Tatsuo Akechi, Tatsuya Morita, Kento Masukawa, Yoshiyuki Kizawa, Satoru Tsuneto, Mitsunori Miyashita

**Affiliations:** 1 Division of Palliative Care and Psycho-Oncology, Nagoya City University Hospital, Nagoya, Japan; 2 Department of Psychiatry and Cognitive-Behavioral Medicine, Graduate School of Medical Sciences and Medical School, Nagoya City University, Nagoya, Japan; 3 Department of Palliative and Supportive Care, Palliative Care Team, and Seirei Hospice, Seirei Mikatahara General Hospital, Hamamatsu, Japan; 4 Research Association for Community Health, Hamamatsu, Japan; 5 Department of Palliative Nursing, Health Sciences, Graduate School of Medicine, Tohoku University, Sendai, Japan; 6 Department of Palliative Medicine, Faculty of Medicine, University of Tsukuba, Tsukuba, Japan; 7 Department of Human Health Sciences, Graduate School of Medicine, Kyoto University, Kyoto, Japan

**Keywords:** Cancer, End-of-life, Delirium, Palliative care, Terminal delirium

## Abstract

**Background:**

We previously developed a 24-item Terminal Delirium-Related Distress Scale (TDDS) to evaluate patient and family distress due to terminal delirium. However, a scale with fewer evaluation items was needed to reduce the burden on terminally ill patients and their families. Thus, the TDDS Shortform (TDDS-SF) was developed, and the validity and reliability of the scale were evaluated.

**Objectives:**

The aim of this study is to evaluate the validity and reliability of TDDS-SF.

**Methods:**

Items with insufficient loading (<0.6) based on factor analysis were removed from the TDDS. Palliative care experts reviewed each item and checked the structure of the scale. Based on their feedback, we developed the TDDS-SF, a 15-item questionnaire consisting of 4 subscales, including “Care for the family,” “Ability to communicate,” “Psychiatric symptoms,” and “Adequate information and discussion about treatment for delirium.” A cross-sectional, self-completed questionnaire survey of bereaved families of cancer patients who were admitted to a hospice/palliative care unit was conducted in August 2018. The survey included the TDDS-SF, Good Death Inventory (GDI), Care Evaluation Scale (CES), and distress score in the Delirium Experience Questionnaire. The validity, including construct validity, convergent validity, discriminant validity, and internal consistency, and reliability, including the Cronbach’s alpha coefficient for internal consistency, of the TDDS-SF were evaluated.

**Results:**

The study included 366 bereaved family members. Factor analysis revealed good construct validity. Convergent validity was demonstrated based on good correlations with the CES (*r* = − 0.54, *P* < 0.001) and the GDI (*r* = − 0.54, *P* < 0.001). Discriminant validity was demonstrated by a low correlation (*r* = 0.23, *P* < 0.001) with the distress scores of bereaved families. The internal consistency was also good (Cronbach’s alpha = 0.70–0.94).

**Significance of results:**

The TDDS-SF is a valid and feasible tool for assessing irreversible terminal delirium-related distress. A study targeting patients and their families with end-of-life delirium is planned for the near future.

## Introduction

Most terminally ill patients experience delirium (Breitbart and Strout 2000; Hosie et al. 2013; Lawlor and Bush 2015; Lawlor et al. 2000; Morita et al. 2001), and the delirium often does not improve before death (de la Cruz et al. 2015; Leonard et al. 2008). The irreversible delirium during the dying process, referred to as terminal delirium (Bush et al. 2014), places a heavy burden on patients, health-care providers, and patients’ families (Cohen et al. 2009; Finucane et al. 2017; Kerr et al. 2013; Partridge et al. 2013). Family caregivers often experience distress in the face of terminal delirium, and the emotional care of family members should be emphasized (Agar 2020). In addition, the standard treatment of terminal delirium has not been established, and family members experience a considerable burden as surrogate decision-makers. A relationship between terminal delirium in patients and depression in bereaved family members was recently demonstrated (Hatano et al. 2022).

Many rating scales are used for delirium (Breitbart et al. 1997; Gaudreau et al. 2005; Inouye et al. 1990; Thurber et al. 2015). However, these scales do not work for patients with terminal delirium who are in poor physical condition and require medication to relieve their distress. In addition, to evaluate terminal delirium, it is necessary to consider a balance between distress relief and communication, between distress relief and the family’ s mental preparation forthe patient’ s death, and between treatment of delirium itself and physical treatment is needed (Uchida et al. 2018). Therefore, specific scales are needed to assess patient and family distress related to terminal delirium.

Based on a previous qualitative analysis (Uchida et al. 2018) and systematic literature search, we conducted a survey regarding the views of bereaved families and developed a questionnaire. Items that bereaved families regarded as important were extracted and an evaluation scale of terminal delirium was developed. This questionnaire was used in a cross-sectional survey of bereaved relatives of cancer patients who were admitted to hospice or palliative care units. Based on this survey, a 24-item Terminal Delirium-Related Distress Scale (TDDS) was developed and validated (Uchida et al. 2021). The TDDS consists of 5 subscales, including support for families and respect for the patient, ability to communicate, hallucinations and delusions, adequate information about the treatment of delirium, and agitation and restlessness.

To reduce the physical and psychological burden of responding to questionnaires on family members and patients, we developed a shortform of the TDDS (TDDS-SF), consisting of 15 items that were most important to bereaved families. The aim of this study was to evaluate the validity and reliability of the TDDS-SF.

## Methods

A nationwide self-administered questionnaire survey of the families of cancer patients who died in hospice/palliative care units certified by the Japan Hospice and Palliative Care Association was conducted. The survey was mailed to the families of patients from the participating facility in August 2018, along with a document explaining the survey. The participants were asked to return the completed questionnaire within 2 weeks. In September 2018, a postcard reminding participants to return the completed questionnaire was sent.

### Participants

Adults from bereaved families of adult patients with cancer who died at participating hospices/palliative care units from February 2014 to January 2018 were included in the study. The exclusion criteria were as follows: (1) family members could not be identified; (2) patient died due to treatment-related death or in the ICU ward; (3) patient used hospices/palliative care unit for <3 days; (4) potential participant was incapable of responding to the self-completed questionnaire due to cognitive impairment, mental disorder, or visual disability; (5) potential participant was not mentally stable; (6) potential participant did not consent to participation; and (7) potential participant’s involvement in the study was undesirable based on a comprehensive judgment made by multiple professionals due to a strong dissatisfaction with or misunderstanding of the medical care received or a poor relationship with medical staff.

### TDDS-SF development

This study was conducted in 2 steps: scale development and scale validation. The TDDS-SF was developed to reduce the burden of answering questionnaires on terminally ill patients and their families. Therefore, the items with insufficient loading (<0.6) in the TDDS were removed based on factor analysis. Palliative care experts, including cancer nurses, palliative care physicians, psycho-oncologists, and clinical psychologists, reviewed each item and checked the structure of the scale. The TDDS-SF consists of 15 items and 4 subscales, including “Care for the family,” “Ability to communicate,” “Psychiatric symptoms,” and “Adequate information and discussion about treatment for delirium.”

### Scale validation

To assess the validity and reliability of the TDDS-SF, a cross-sectional, self-completed questionnaire survey of bereaved families of cancer patients who were admitted to a hospice/palliative care unit was conducted. The validity assessment included construct validity, convergent validity, and discriminant validity, and the reliability was assessed using Cronbach’s alpha coefficient for internal consistency.

### Good Death Inventory – Short Version

The distress caused by terminal delirium is related to achieving a good death (Uchida et al. 2021). Thus, the convergent validity between the Good Death Inventory (GDI) and TDDS-SF was assessed. The short version of the previously validated GDI was used to evaluate the patient’s achievement of a good death from the perspective of the bereaved family (Miyashita et al. 2008). The short version of the GDI includes 18 representative items, which are evaluated using a 7-point Likert scale. The total score is calculated by summing the scores for all attributes; a high total score indicates the achievement of a good death.

### Care Evaluation Scale – Short Version

The distress caused by terminal delirium is related to end-of-life care (Wright et al. 2014). Therefore, we examined the convergent validity between the end-of-life care and the TDDS-SF. End-of-life care was assessed using the revised short version of the Care Evaluation Scale (CES2) (Miyashita et al. 2017). The CES2 evaluates end-of-life care from the perspective of bereaved family members, focusing on the structure and process of care. The validity and reliability of the CES2e has been confirmed. The CES2 consists of 10 representative items. The questions allow respondents to evaluate the necessity for improvement for each item on a 6-point Likert-type scale. The total score was 100 points, with higher scores indicating good structure or process of care.

### Delirium Experience Questionnaire

The Delirium Experience Questionnaire (DEQ) (Breitbart et al. 2002) was developed to assess recall of the delirium experience and the degree of distress related to the delirium episode experienced by patients, spouses/caregivers, and nurses. The DEQ includes 6 questions for patients who have recovered from an episode of delirium. In addition, 1 question each is directed to the spouses/caregivers and nurses. The following question for the bereaved family members was used in our assessment: “How distressed were you during the patient’s delirium?” Answers were given on a 5-point scale from 0 to 4. Because terminal delirium cannot be adequately evaluated using general delirium assessment scales (Leonard et al. 2014), the 2 were compared for discriminant validity.

### Statistical methods

The TDDS-SF was developed using factor analysis followed by varimax rotation. The number of factors was determined using the Scree test. The assessment of construct validity was based on how well a repetition of the factor analysis reproduced the factor loading pattern in the phase of scale development. Convergent validity was examined by calculating Pearson’s correlations between the TDDS-SF and the CES and GDI scores. Discriminant validity was examined using Pearson’s correlations between the TDDS scores and the DEQ distress score of the bereaved family. The internal consistency was assessed using the Cronbach’s alpha coefficient. All statistical analyses were conducted using SPSS statistical software (version 24; IBM Corporation, Armonk, NY, USA).

## Results

### Development of the Terminal Delirium-Related Distress Scale – Short Form

The 15-item draft questionnaire was based on the 24-item TDDS. The responses were evaluated by factor analysis followed by varimax rotation. Four factors were identified by the Scree test. The first subscale consisted of 5 items related to care and support for the family (“Care for the family” subscale). The second subscale consisted of 3 items related to communication (“Ability to communicate” subscale). The third subscale consisted of 4 items related to hallucinations, delusions, and agitation (“Psychiatric symptoms” subscale), and the fourth subscale consisted of 3 items related to the explanation and discussion about terminal delirium (“Adequate information and discussion about the treatment of delirium” subscale). An English version of the complete scale is presented in Appendix 1.

### Validation of the TDDS-SF

Of the 1,710 families receiving surveys, 1,112 (66%) bereaved family members returned the surveys, including 867 (51%) completed responses, and 366 bereaved family members (42% of the responders) indicated that their loved one had experienced delirium. Participant characteristics are shown in Table 1.

**Table 1.** Bereaved families’ and patients’ characteristics (*N* = 366)

Bereaved family		*N*	%
Age	Mean 59.3y (SD: 12.4)		
	Median 59y (range: 26−93)		
Sex	Female	244	67
Relation to the deceased	Spouse	151	42
	Child	157	43
	Son-in-law・Daughter-in-law	11	3
	Parent	5	1
	Sibling	27	8
	Other	11	3
Education	≥12 years	194	54
Patient		*N*	%
Marital status	Married/partnered	225	62
	Unmarried	28	8
	Bereaved	84	23
	Divorced	24	7
Living with someone	Yes	271	76

The results of the factor analysis are shown in Table 2. Repeated factor analysis revealed that the construct validity was good.

**Table 2.** Factor validity of the terminal delirium related distress scale-short form, optimal 4 domains (*N* = 358)

	Factor			
	Care for the family	Ability to communicate	Psychiatric symptoms	Adequate information and discussion about treatment for delirium
9. Consideration was given not to make caring a patient too heavy a burden on families.	0.94	−0.02	−0.05	0.07
8. Medical staff provided emotional support for family.	0.85	0.07	0	−0.001
11. Medical staff was present with family when they felt uneasy.	0.71	−0.03	0.02	−0.11
10. Medical staff coached families what they could do for patients.	0.68	−0.005	−0.009	−0.24
12. Medical staff responded promptly as needed.	0.47	−0.04	0.11	−0.27
6. Patients were able to communicate even if they took anxiolytic or hypnotic.	0.02	0.89	−0.001	−0.005
5. Patients were able to communicate even if delirium did not obtain complete remission.	−0.1	0.81	−0.01	−0.11
7. Patients continued to be what the patient was.	0.1	0.78	0.007	−0.004
4. Patients had delusion.^a^	−0.12	−0.14	0.75	−0.12
3. Patients had hallucination.^a^	0.01	−0.18	0.73	−0.03
2. Patients were excited and agitated.^a^	0.01	0.08	0.66	0.03
1. Patients were restless.^a^	0.05	0.12	0.44	0.12
14. Family could sufficiently discuss about the treatment plan with health professionals.	0.07	0.12	0.03	−0.85
13. Health-care professional explained adequately about the treatment plan and future perspective.	0.22	0.02	0.007	−0.78
15. Health-care professional explained adequately about the nature of delirium and reasons why the delirium occurs.	0.17	0.1	−0.11	−0.74

a Reverse item.

The convergent validity, assessed by the correlation between the TDDS-SF and the CES2 (*r* = −0.54, *P* < 0.001) and GDI (*r* = −0.54, *P* < 0.001), was good. Discriminant validity was confirmed by the poor correlation between the TDDS-SF and the distress scores of the bereaved family members on the DEQ (*r* = 0.23, *P* < 0.001) (Table 3).

**Table 3.** Correlation between Terminal Delirium-Related Distress Scale – Short Form and CES・DEQ

Subscale	CES	GDI	DEQ
Care for the family	**−0.58**	**−0.49**	**0.15**
Ability to communicate	**−0.22**	**−0.26**	**0.19**
Psychiatric symptoms	−0.04	**−0.17**	**0.16**
Adequate information and discussion about treatment for delirium	**−0.49**	**−0.41**	**0.18**
Total score	**−0.54**	**−0.54**	**0.23**

CES = Care Evaluation Scale; GDS = Good Death Inventory; DEQ: Distress score of bereaved family in Delirium Experience Questionnaire.

Bold: *P* < 0.001.

The TDDS-SF showed good internal consistency (Cronbach’s alpha coefficient for all 15 items = 0.82) (Table 4).

**Table 4.** Reliability of Terminal Delirium-Related Distress Scale – Short Form

Subscale	Mean	SD	Cronbach’s α
Care for the family	11.2	3.31	0.9
Ability to communicate	6.6	1.92	0.86
Psychiatric symptoms	9.44	2.81	0.7
Adequate information and discussion about treatment for delirium	6.95	2.43	0.94
Total score	34.2	6.68	0.82

SD = standard deviation.

## Discussion

To reduce the burden on terminally ill patients and their families, a shortened version of the TDDS was developed. The original version of the TDDS consists of 24 items. To make this scale easier to use in clinical practice, a 15-item version of the TDDS was created by extracting important items based on the perspective of the bereaved family.

Factor analysis confirmed the scale’s structure. The analysis also indicated that appropriate treatment and care for terminal delirium consists of 4 dimensions: care for the family, ability to communicate, psychiatric symptoms, and adequate information and discussion about the treatment of delirium.

Convergent validity was confirmed by the significant correlations between the 3 subscales of the TDDS-SF and the CES. The subscales of “Care for the family” and “Adequate information and discussion about treatment for delirium” moderately correlated with the total CES score because these 2 subscales are related to the structure and process of care, which are assessed by the CES. The “Psychiatric symptom” subscale did not significantly correlate with the CES because this subscale pertains to symptoms rather than the structure and process of care.

Convergent validity was also demonstrated by the significant correlations between the 4 subscales of the TDS and the GDI. The “Care for the family” and “Adequate information and discussion about treatment for delirium” subscales moderately correlated with the total GDI scores because the content assessed by these 2 subscales is necessary for a good death. The “Ability to communicate” subscale only slightly correlated with the total GDI score because the GDI-short version excluded items on communication. The “Psychiatric symptoms” subscale did not closely correlate with the total GDI score because the short version of the GDI excludes psychiatric symptoms.

Discriminant validity was shown by significant correlations between the 2 subscales of the TDDS and the DEQ.

All subscales of the TDDS-SF slightly correlated with the DEQ. The DEQ assesses the degree of distress related to a reversible delirium episode and includes only the distress of family caregivers. The TDDS-SF specifically targets terminal delirium and evaluates the overall severity of distress caused by terminal delirium, including family caregivers and the patient. Therefore, all of the TDDS-SF subscales significantly correlated with the DEQ. However, the association was weak (Table 3).

Each of the 4 subscales and the total score of the TDDS-SF showed high internal consistency. Due to the specific nature of the target population, we did not assess test–retest reliability.

The development of this scale began with an exploration of the best treatment and care for patients experiencing terminal delirium and their families. The TDDS-SF was developed with the help of family members who were bereaved after their loved ones experienced terminal delirium, not terminal delirium patients and their families who were already in a state of extreme distress.

There are several limitations to this study. First, the questionnaire was not administered immediately after the patient’s death but after some time had passed. Thus, recall bias may have occurred, and the level of distress may vary depending on the time since the bereavement. Second, these results may be difficult to generalize because the study targeted families of patients who died in hospice or palliative care wards. Third, the response rate was 66%. However, only 42% of the respondents represented bereaved families who had experienced terminal delirium. The study was not considered fatally flawed for this reason. Fourth, the subjects were all Japanese, and cross-cultural validation has not been conducted. Fifth, criterion-related validity could not be assessed because no gold standard for terminal delirium has been established.

In conclusion, our results show that the TDDS-SF is a valid and reliable scale for assessing the 4 aspects of terminal delirium care and treatment for bereaved family members. This scale, similar to the original version, may help healthcare providers to provide patients and families with appropriate care and treatment for terminal delirium.

## Supporting information

Uchida et al. supplementary material Uchida et al. supplementary material

## Supplementary material

The supplementary material for this article can be found at https://doi.org/10.1017/S1478951525000227.

## References

[ref1] Agar MR (2020) Delirium at the end of life. *Age and Ageing.* 49(3), 337–340. doi:10.1093/ageing/afz171 31925413

[ref2] Breitbart W, Gibson C and Tremblay A (2002) The delirium experience: Delirium recall and delirium-related distress in hospitalized patients with cancer, their spouses/caregivers, and their nurses. *Psychosomatics* 43(3), 183–194. doi:10.1176/appi.psy.43.3.183 12075033

[ref3] Breitbart W, Rosenfeld B, Roth A, et al. (1997) The Memorial Delirium Assessment Scale. *Journal of Pain and Symptom Management* 13(3), 128–137. doi:10.1016/s0885-3924(96)00316-8 9114631

[ref4] Breitbart W and Strout D (2000) Delirium in the terminally ill. *Clinics in Geriatric Medicine* 16(2), 357–372. doi:10.1016/s0749-0690(05)70061-6 10783433

[ref5] Bush SH, Leonard MM, Agar M, et al. (2014) End-of-life delirium: Issues regarding recognition, optimal management, and the role of sedation in the dying phase. *Journal of Pain and Symptom Management* 48(2), 215–230. doi:10.1016/j.jpainsymman.2014.05.009 24879997

[ref6] Cohen MZ, Pace EA, Kaur G, et al. (2009) Delirium in advanced cancer leading to distress in patients and family caregivers. *Journal of Palliative Care* 25(3), 164–171. 10.1177/082585970902500303 19824277

[ref7] de la Cruz M, Ransing V, Yennu S, et al. (2015) The frequency, characteristics, and outcomes among cancer patients with delirium admitted to an acute palliative care unit. *The Oncologist* 20(12), 1425–1431. doi:10.1634/theoncologist.2015-0115 26417036 PMC4679079

[ref8] Finucane AM, Lugton J, Kennedy C, et al. (2017) The experiences of caregivers of patients with delirium, and their role in its management in palliative care settings: An integrative literature review. *Psycho-Oncology* 26(3), 291–300. doi:10.1002/pon.4140 27132588 PMC5363350

[ref9] Gaudreau JD, Gagnon P, Harel F, et al. (2005) Fast, systematic, and continuous delirium assessment in hospitalized patients: The nursing delirium screening scale. *Journal of Pain and Symptom Management* 29(4), 368–375. doi:10.1016/j.jpainsymman.2004.07.009 15857740

[ref10] Hatano Y, Morita T, Mori M, et al. (2022) Association between experiences of advanced cancer patients at the end of life and depression in their bereaved caregivers. *Psycho-Oncology* 31(7), 1243–1252. doi:10.1002/pon.5915 35253947

[ref11] Hosie A, Davidson PM, Agar M, et al. (2013) Delirium prevalence, incidence, and implications for screening in specialist palliative care inpatient settings: A systematic review. *Palliative Medicine* 27(6), 486–498. doi:10.1177/0269216312457214 22988044

[ref12] Inouye SK, van Dyck Ch, Alessi CA, et al. (1990) Clarifying confusion: The confusion assessment method. A new method for detection of delirium. *Annals of Internal Medicine.* 113(12), 941–948. doi:10.7326/0003-4819-113-12-941 2240918

[ref13] Kerr CW, Donnelly JP, Wright ST, et al. (2013) Progression of delirium in advanced illness: A multivariate model of caregiver and clinician perspectives. *Journal of Palliative Medicine* 16(7), 768–773. doi:10.1089/jpm.2012.0561 23718872

[ref14] Lawlor PG and Bush SH (2015) Delirium in patients with cancer: Assessment, impact, mechanisms and management. *Nature Reviews Clinical Oncology* 12(2), 77–92. doi:10.1038/nrclinonc.2014.147 25178632

[ref15] Lawlor PG, Gagnon B, Mancini IL, et al. (2000) Occurrence, causes, and outcome of delirium in patients with advanced cancer: A prospective study. *Archives of Internal Medicine* 160(6), 786–794. doi:10.1001/archinte.160.6.786 10737278

[ref16] Leonard MM, Agar M, Spiller JA, et al. (2014) Delirium diagnostic and classification challenges in palliative care: Subsyndromal delirium, comorbid delirium-dementia, and psychomotor subtypes. *Journal of Pain and Symptom Management* 48(2), 199–214. doi:10.1016/j.jpainsymman.2014.03.012 24879995

[ref17] Leonard M, Raju B, Conroy M, et al. (2008) Reversibility of delirium in terminally ill patients and predictors of mortality. *Palliative Medicine* 22(7), 848–854. doi:10.1177/0269216308094520 18755829

[ref18] Miyashita M, Aoyama M, Nakahata M, et al. (2017) Development the Care Evaluation Scale Version 2.0: A modified version of a measure for bereaved family members to evaluate the structure and process of palliative care for cancer patient. *BMC Palliative Care* 16(1), 8. doi:10.1186/s12904-017-0183-2 PMC5259912 28114917

[ref19] Miyashita M, Morita T, Sato K, et al. (2008) Good death inventory: A measure for evaluating good death from the bereaved family member’s perspective. *Journal of Pain and Symptom Management* 35(5), 486–498. doi:10.1016/j.jpainsymman.2007.07.009 18358685

[ref20] Morita T, Tei Y, Tsunoda J, et al. (2001) Underlying pathologies and their associations with clinical features in terminal delirium of cancer patients. *Journal of Pain and Symptom Management* 22(6), 997–1006. 10.1016/S0885-3924(01)00360-8 11738162

[ref21] Partridge JS, Martin FC, Harari D, et al. (2013) The delirium experience: What is the effect on patients, relatives and staff and what can be done to modify this? *International Journal of Geriatric Psychiatry* 28(8), 804–812. doi:10.1002/gps.3900 23112139

[ref22] Thurber S, Kishi Y, Trzepacz PT, et al. (2015) Confirmatory factor analysis of the Delirium Rating Scale Revised-98 (DRS-R98). *The Journal of Neuropsychiatry and Clinical Neurosciences* 27(2), e122–127. doi:10.1176/appi.neuropsych.13110345 25923855

[ref23] Uchida M, Akechi T, Morita T, et al. (2021) Development and validation of the Terminal Delirium-Related Distress Scale to assess irreversible terminal delirium. *Palliative and Supportive Care* 19(3), 287–293. doi:10.1017/S1478951520001340 33397541

[ref24] Uchida M, Morita T, Ito Y, et al. (2018) Goals of care and treatment in terminal delirium: A qualitative study of the views and experiences of healthcare professionals caring for patients with cancer. *Palliat Support Care* 17(4), 403–408. doi:10.1017/S1478951518000780 30466502

[ref25] Wright DK, Brajtman S and Macdonald ME (2014) A relational ethical approach to end-of-life delirium. *Journal of Pain and Symptom Management* 48(2), 191–198. doi:10.1016/j.jpainsymman.2013.08.015 24417807

