# Supplementary material for: Development and validation of the Terminal Delirium-Related Distress Scale – Shortform
Source: Palliat Support Care. 2025 Mar 14;23:e78. doi: 10.1017/S1478951525000227 (PMC13166458; doi:10.1017/S1478951525000227)
Supplement: Uchida et al. supplementary material [file S1478951525000227sup001.docx]

| When patients were delirious, did you see any of the following? Please circle one of the following that applies from “strongly agree” to “strongly disagree”. | Strongly agree | Agree | Disagree | Strongly disagree |
| --- | --- | --- | --- | --- |
| **Distress of patients** |  |  |  |  |
| 1. Patients were restless. * | 1 | 2 | 3 | 4 |
| 1. Patients were excited and agitated. * | 1 | 2 | 3 | 4 |
| 1. Patients had hallucination. * | 1 | 2 | 3 | 4 |
| 1. Patients had delusion. * | 1 | 2 | 3 | 4 |
| **Communication with patients** |  |  |  |  |
| 1. Patients were able to communicate even if delirium did not obtain complete remission. | 1 | 2 | 3 | 4 |
| 1. Patients were able to communicate even if they took anxiolytic or hypnotic. | 1 | 2 | 3 | 4 |
| 1. Patients continued to be what the patient was. | 1 | 2 | 3 | 4 |
| **Support and explanation of health care providers** |  |  |  |  |
| 1. Health care providers provided emotional support for family. | 1 | 2 | 3 | 4 |
| 1. Consideration was given not to make caring a patient too heavy a burden on families. | 1 | 2 | 3 | 4 |
| 1. Health care providers coached families what they could do for patients. | 1 | 2 | 3 | 4 |
| 1. Health care providers was present with family when they felt uneasy. | 1 | 2 | 3 | 4 |
| 1. Health care providers responded promptly as needed. | 1 | 2 | 3 | 4 |
| 1. Health care providers explained adequately about the treatment plan and future perspective. | 1 | 2 | 3 | 4 |
| 1. Family could sufficiently discuss about the treatment plan with health care providers. | 1 | 2 | 3 | 4 |
| 1. Health care providers explained adequately about the nature of delirium and reasons why the delirium ocurrs. | 1 | 2 | 3 | 4 |

*Reverse item
